# Aluminum–Nitrogen Interactions in the Soil–Plant System

**DOI:** 10.3389/fpls.2018.00807

**Published:** 2018-06-18

**Authors:** Xue Q. Zhao, Ren F. Shen

**Affiliations:** ^1^State Key Laboratory of Soil and Sustainable Agriculture, Institute of Soil Science, Chinese Academy of Sciences, Nanjing, China; ^2^University of Chinese Academy of Sciences, Beijing, China

**Keywords:** aluminum, nitrogen, ammonium, nitrate, interaction, plant, acid soil

## Abstract

Aluminum (Al) is the most abundant metal in the Earth’s crust and is not an essential element for plant growth. In contrast, nitrogen (N) is the most important mineral element for plant growth, but this non-metal is often present at low levels in soils, and plants are often N deficient. Aluminum toxicity is dominant in acid soils, and so plants growing in acid soils have to overcome both Al toxicity and N limitation. Because of low N-use efficiency, large amounts of N fertilizers are applied to crop fields to achieve high yields, leading to soil acidification and potential Al toxicity. Aluminum lowers plant N uptake and N-use efficiency because Al inhibits root growth. Although numerous studies have investigated the interactions between Al and N, a complete review of these studies was lacking. This review describes: (1) the link between plant Al tolerance and ammonium/nitrate (NH_4_^+^/NO_3_^-^) preference; (2) the effects of NH_4_^+^/NO_3_^-^ and pH on Al toxicity; (3) the effects of Al on soil N transformations; and (4) the effects of Al on NH_4_^+^/NO_3_^-^ uptake and assimilation by plants. Acid soils are characterized chemically by a relatively high ratio of NH_4_^+^ to NO_3_^-^ and high concentrations of toxic Al. Aluminum-tolerant plants generally prefer NH_4_^+^ as an N source, while Al-sensitive plants prefer NO_3_^-^. Compared with NO_3_^-^, NH_4_^+^ increases the solubilization of toxic Al into soil solutions, but NH_4_^+^ generally alleviates Al phytotoxicity under solution culture because the protons from NH_4_^+^ compete with Al^3+^ for adsorption sites on the root surface. Plant NO_3_^-^ uptake and nitrate reductase activity are both inhibited by Al, while plant NH_4_^+^ uptake is inhibited to a smaller degree than NO_3_^-^. Together, the results of numerous studies indicate that there is a synergistic interaction between plant Al tolerance and NH_4_^+^ nutrition. This has important implications for the adaptation of plants to acid soils that are dominated chemically by toxic Al as well as NH_4_^+^. Finally, we discuss how this knowledge can be used to increase plant Al tolerance and N-use efficiency in acid soils.

## Introduction

Acid soils cover approximately 30% of the ice-free land and up to 70% of potentially arable soils worldwide (von Uexküll and Mutert, 1995). Acid soils occur mainly in humid tropical and temperate areas (von Uexküll and Mutert, 1995), where water and heat are generally abundant for plant growth, implying that acid soils have huge productive potential. However, plant productivity in acid soils is limited primarily by aluminum (Al) toxicity accompanied by deficiencies of some nutrients (Zhao et al., 2014). The improvement of crop productivity in acid soils depends on the dual enhancement of plant Al tolerance and nutrient-use efficiency.

Nitrogen (N) is the most abundant mineral nutrient required by plants. Soil N availability greatly affects the growth and development of crops worldwide (Gutiérrez, 2012). Nitrogen deficiency is a widespread problem for plants grown in terrestrial ecosystems (Vitousek and Howarth, 1991), and it is also a major factor limiting plant growth in acid soils (Fageria and Baligar, 2001). Large amounts of N fertilizers are used in agriculture to grow crops that feed an increasing global population every year. Erisman et al. (2008) estimated that N fertilizer has supported around 4 billion people born since 1908, accounting for approximately 27% of the world’s population over the past century. At the same time, excess N fertilization is causing environmental problems such as water eutrophication, greenhouse gas emissions, nitrate (NO_3_^-^) loss, acid rain, and soil acidification due to low N-use efficiency (Ju et al., 2009). High yields and high nutrient-use efficiency are essential for contemporary agriculture. Therefore, there is an urgent need to increase plant N-use efficiency by understanding the responses to N (Kant et al., 2011).

Aluminum is the most abundant metal in the Earth’s crust. It is not an essential element for plants, and excess Al is toxic to most plants. The primary symptom of Al phytotoxicity is the inhibition of root elongation, which can occur after exposure to Al^3+^ at concentrations as low as μM levels within 1 h (Matsumoto, 2000; Kochian et al., 2005; Ma, 2007). This inhibition can be caused by reductions in cell elongation and cell division, which are attributed to Al interference with the cell wall, plasma membrane, the cytoskeleton, oxidative stress, signal transduction pathways, cytoplasm calcium homeostasis, magnesium uptake, and auxin polar transport (Ma, 2007). Plants have two strategies to detoxify Al (Ma, 2007). One is to exclude Al from the root tips (exclusion mechanism) and the other is to tolerate Al that enters the plant body (internal tolerance mechanism). Roots are the main organ for plants to take up nutrients from the growth medium, so Al toxicity inevitably affects the ability of plants to acquire nutrients from acid soils. On one hand, the inhibitory effects of Al on root growth can reduce the amounts of nutrients taken up by plants because of the small root volume. On the other hand, Al may directly affect the transport and metabolism of nutrients within plants. Interactions between Al and many nutrients often occur within soils and plants (Zhao et al., 2014). Most reports have focused on the effects of various externally added nutrients on Al phytotoxicity (Zhao et al., 2014), but the effects of Al on the uptake of these nutrients by plants and their corresponding mechanisms have received relatively little attention.

Aluminum is beneficial and even potentially essential for some plant species (Bojórquez-Quintal et al., 2017), because of the Al-induced stimulation of nutrient uptake (Watanabe and Osaki, 2002). Aluminum supply was shown to stimulate N uptake by several plant species adapted to acid soils (Osaki et al., 1997), and Al treatments increased shoot N contents in wheat and rye (Dinev and Stancheva, 1993). In contrast, Al reduced root N uptake and its upward translocation to shoots in sorghum and corn (Gomes et al., 1985; Pintro et al., 1996). Aluminum promoted the growth of plants supplied with ammonium (NH_4_^+^) but inhibited that of plants supplied with NO_3_^-^ (Zhao et al., 2014). Nitrogen is a metabolic element involved in the synthesis of amino acids and proteins within plants. Knowledge about Al–N interactions may supply new information to explain instances where Al benefits plant growth.

Several reviews have focused on the interactions between Al and phosphorus (Chen et al., 2012), calcium (Rengel and Zhang, 2003; Meriño-Gergichevich et al., 2010), magnesium (Bose et al., 2011; Chen and Ma, 2013), boron, and silicon (Hodson and Evans, 1995; Horst et al., 2010). Aluminum is a metal and a toxic element to many plants, while N is a non-metal and is an essential element for all plants. More than 100 papers have reported on Al–N interactions so far, highlighting the importance of this topic. Despite the large amount of literature on Al–N interactions, there has been no systematic review of this topic so far. Here, we provide a detailed description and analysis of studies on the interactions between Al and N, including the link between plant Al tolerance and NH_4_^+^/NO_3_^-^ preference, the effects of NH_4_^+^/NO_3_^-^ and pH on Al toxicity, the effects of Al on soil N transformations, and the effects of Al on NH_4_^+^/NO_3_^-^ uptake and assimilation. We also propose a strategy for improving plant Al tolerance and N-use efficiency in acid soils.

## Link Between Plant Al Tolerance and Inorganic N Preference

Acid soils are characterized by poor nitrification and high levels of soluble Al, while neutral to calcareous soils show high nitrification and lower levels of Al toxicity (Zhao et al., 2014; Che et al., 2015). The two main inorganic N sources available for plant growth are NH_4_^+^ and NO_3_^-^. Therefore, on the basis of the environment driving evolution, plants originating from acid soils are Al tolerant and prefer NH_4_^+^ to NO_3_^-^, while those originating from neutral to calcareous soils are Al sensitive and prefer NO_3_^-^ to NH_4_^+^ (Gigon and Rorison, 1972; Foy and Fleming, 1978; Rorison, 1985; Falkengren-Grerup, 1995; Marschner, 1995; Maathuis, 2009; Zhao et al., 2013b) (**Table 1**). For instance, the growth of lowbush blueberry, which is adapted to strongly acid soils, was shown to be greatly promoted by NH_4_^+^ but strongly inhibited by NO_3_^-^ (Townsend, 1966; Townsend and Blatt, 1966). Wheat and barley are Al-sensitive and prefer NO_3_^-^ (Malhi et al., 1988; Cramer and Lewis, 1993; Famoso et al., 2010), while tea and rice are Al-tolerant and prefer NH_4_^+^ (Ruan et al., 2007; Famoso et al., 2010; Zhao et al., 2013b). The activity of NO_3_^-^ reductase could not be detected in some calcifuge species, suggesting that they have a restricted ability to utilize NO_3_^-^ (Havill et al., 1974). Rice (*Oryza sativa*) has two subspecies, *indica* and *japonica*. *Indica* rice cultivars generally prefer NO_3_^-^, while *japonica* cultivars prefer NH_4_^+^ (Zhao et al., 2013b; Hu et al., 2015). Correspondingly, *indica* rice cultivars are generally Al sensitive, while *japonica* cultivars are Al tolerant (Zhao et al., 2013b). Among different rice cultivars, Al tolerance is closely related to NH_4_^+^ and NO_3_^-^ preference (Zhao et al., 2013b).

**Table 1:** Aluminum tolerance and NH_4_^+^/NO_3_^-^ preference of plant species.

Taxon	Al tolerance	NH_4_^+^/NO_3_^-^ preference	Reference
*Vaccinium angustifolium*	Tolerant	NH_4_^+^	Townsend, 1966; Townsend and Blatt, 1966
*Deschampsia flexuosa*	Tolerant	NH_4_^+^	Rorison, 1985
*Oxalis acetosella, Carex pilulifera, Festuca gigantea, Poa nemoralis, Deschampsia flexuosa, Stellaria holostea, Rumex acetosella*	Tolerant	NH_4_^+^	Falkengren-Grerup, 1995
*Camellia sinensis*	Tolerant	NH_4_^+^	Ruan et al., 2007
*Oryza sativa* subsp. *japonica*	Tolerant	NH_4_^+^	Zhao et al., 2013b
			
*Holcus lanatus, Bromus erectus*	Sensitive	NO_3_^-^	Rorison, 1985
*Hordeum vulgare*	Sensitive	NO_3_^-^	Malhi et al., 1988
*Triticum aestivum*	Sensitive	NO_3_^-^	Cramer and Lewis, 1993; Famoso et al., 2010
*Urtica dioica, Ficaria verna, Melandrium rubrum, Aegopodium podagraria, Geum urbanum, Bromus benekenii, Sanguisorba minor, Melica ciliata, Silene rupestris, Viscaria vulgaris, Plantago lanceolata*	Sensitive	NO_3_^-^	Falkengren-Grerup, 1995
*Oryza sativa* subsp. *indica*	Sensitive	NO_3_^-^	Zhao et al., 2013b


The above analyses collectively suggest that Al-tolerant plant species and genotypes utilize NH_4_^+^ more efficiently than NO_3_^-^ (**Table 1**). This knowledge is helpful for the selection of crop genotypes with both high Al tolerance and N-use efficiency via breeding or genetic modification. The selection of such genotypes should reduce the amount of N fertilizer required and improve plant growth in acid soils. However, the molecular mechanism underlying the link between plant Al tolerance and inorganic N preference is unclear. The two characteristics of grain protein content and acidity tolerance were found to be positively correlated among different wheat lines (Mesdag et al., 1970). In addition, a quantitative trait locus genetic analysis revealed that loci associated with Al tolerance and NH_4_^+^ utilization were located in similar regions of rice genome (Ogawa et al., 2014). An important goal for future research is to uncover the mechanism of the link between plant Al tolerance and inorganic N preference at the molecular and genetic levels.

## Effects of NH_4_^+^, NO_3_^-^, and pH on Al Tolerance

**FIGURE 1:**
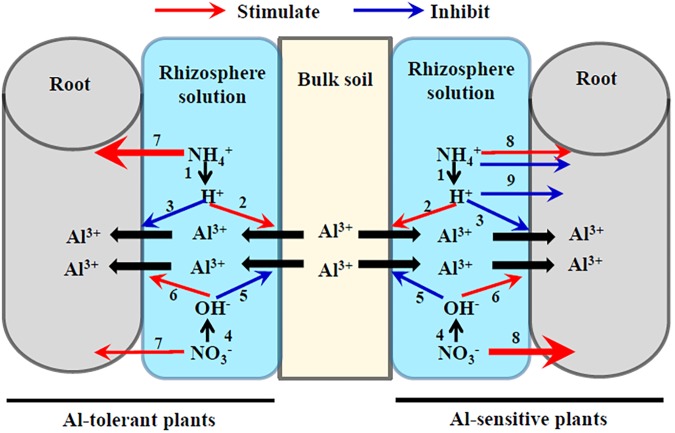
Schematic diagram of possible effects of NH_4_^+^ and NO_3_^-^ on the adsorption and desorption of Al on the root–soil interface. NH_4_^+^ acidifies rhizosphere solution (1), which stimulates the desorption of Al from bulk soils into rhizosphere solution (2) but inhibits the adsorption of Al from rhizosphere solutions to plant roots (3) both because of the competition between Al^3+^ and H^+^. In contrast, NO_3_^-^ alkalizes rhizosphere solution (4), which inhibits the desorption of Al from soils into rhizosphere solution (5) but stimulates the adsorption of Al from rhizosphere solutions to plant roots (6) because NO_3_^-^-increased negative electrical charge of root surface. Al-tolerant plant species prefer NH_4_^+^ to NO_3_^-^ (7), while Al-sensitive plant species prefer NO_3_^-^ to NH_4_^+^ (8). Excess NH_4_^+^ and H^+^ are both toxic to the growth of Al-sensitive plant species (9). Consequently, NH_4_^+^ alleviates Al toxicity to Al-tolerant plant species while aggravates Al toxicity to Al-sensitive plant species compared with NO_3_^-^.

In recent decades, various anthropogenic activities have greatly accelerated soil acidification in Chinese crop fields (Guo et al., 2010; Liang et al., 2013). Among these activities is the excess use of NH_4_^+^ fertilizer (Barak et al., 1997; Fang et al., 2014). Atmospheric NH_4_^+^ deposition is also an important factor resulting in soil acidification (van Breemen et al., 1982). Nitrification is the mechanism by which NH_4_^+^ acidifies soils. During the nitrification of NH_4_^+^ to NO_3_^-^, H^+^ are released into soils, which increase the concentration of soluble Al (van Breemen et al., 1982; Mulder et al., 1989; Mulder and Stein, 1994; Che et al., 2015) (**Figure 1**). Thus, NH_4_^+^ facilitates the occurrence of Al toxicity much more than NO_3_^-^ does. However, increased soluble Al content in soils caused by low pH does not always increase Al phytotoxicity, because lower pH can result in the desorption of Al from plant roots into the rhizosphere solution (**Figure 1**).

Early studies showed that changes in root zone pH due to ion uptake imbalances were related to Al tolerance in triticale, wheat, and rye under certain solution and soil conditions (Mugwira and Patel, 1977). The plant growth medium can be acidified due to NH_4_^+^ uptake by plant roots and the nitrification of NH_4_^+^ to NO_3_^-^. Alternatively, the growth medium can be alkalinized due to the uptake of NO_3_^-^ by plant roots. Because Al toxicity occurs in acid soils, one could speculate that the preferential utilization of NO_3_^-^ relative to NH_4_^+^ can enhance plant Al tolerance through increasing the pH of the growth medium via NO_3_^-^ uptake. The Al tolerance of some wheat varieties was attributable to their abilities to preferentially utilize NO_3_^-^ relative to NH_4_^+^ through rhizosphere alkalization (Foy et al., 1965, 1967; Foy and Fleming, 1978, 1982; Fleming, 1983; Taylor and Foy, 1985a,b,c). The results of subsequent studies, however, indicated that genotypic differences in wheat Al tolerance were not caused by differences in rhizosphere pH induced by the differential uptake of NH_4_^+^ and NO_3_^-^ (Taylor, 1988a,b; Miyasaka et al., 1989). Instead, the differences in the uptake of NH_4_^+^ and NO_3_^-^ among different wheat genotypes were suggested to be the result of, rather than the cause of, differences in A1 tolerance among genotypes (Taylor, 1988a,b; Miyasaka et al., 1989). Another research demonstrated that the decrease in the growth medium pH under Al stress was greater for an Al-tolerant wheat genotype than an Al-sensitive one (Ikeda and Yamanishi, 1999). Therefore, genotypic differences in the relative Al tolerance of wheat could not be explained by root-induced pH changes due to the uptake of NH_4_^+^ and NO_3_^-^.

Three reports on rice plants drew different conclusions. In two studies, an Al-tolerant rice genotype had a stronger ability than an Al-sensitive genotype to increase nutrient solution pH through efficient NO_3_^-^ uptake and metabolism (Ganesan et al., 1993; Justino et al., 2006). However, another study (van Hai et al., 1989) obtained the opposite result, in that an Al-resistant genotype took up more NH_4_^+^ and acidified the nutrient solution to a greater degree than did an Al-sensitive one. In barley, Al tolerance of different cultivars was not related to the root-induced pH change by the uptake of inorganic N sources from the growth medium (Wagatsuma and Yamasaku, 1985). Similarly, differences in pH changes in the growth medium were not related to differences in A1 tolerance between two sorghum genotypes (Galvez and Clark, 1991). In fact, the NO_3_^-^ uptake rate was found to be higher in an Al-sensitive sorghum genotype than in an Al-tolerant one (Cambraia et al., 1989). Genotypic differences in the Al tolerance of soybean plants were not associated with the difference in NH_4_^+^ uptake vs. NO_3_^-^ uptake and root-induced pH changes (Klotz and Horst, 1988b). Changes in the medium pH were also not related to Al tolerance in triticale (Antunes and Antonieta Nunes, 1997). These analyses further demonstrated that genotypic differences in the Al tolerance of diverse plant species cannot be explained only by root-induced pH changes due to NH_4_^+^ and NO_3_^-^ uptake.

Since low pH increases the concentrations of soluble Al in soils, the alkalization of the rhizosphere was proposed to be an important mechanism of plant Al tolerance (Matsumoto, 2000; Kochian et al., 2004; Ma, 2007). However, several studies demonstrated that H^+^ could alleviate Al toxicity because H^+^ competed with Al^3+^ for adsorption to the root surface (Kinraide et al., 1992; Godbold et al., 1995; Zhao et al., 2009; Zhao et al., 2014). A supply of H^+^ also alleviated Al toxicity in bacteria (Kinraide and Sweeney, 2003) and yeast (Zhao et al., 2017). These results implied that Al toxicity is much lower at low pH than at high pH under a certain acid pH range (pH < 5.0) because of the H^+^ alleviation of Al phytotoxicity. The uptake of NH_4_^+^ and NO_3_^-^ decreases and increases the pH of the medium, respectively. Many reports have indicated that NH_4_^+^ supply can enhance plant Al tolerance, while NO_3_^-^ supply aggravates Al toxicity (**Table 2**). In some studies, Al was found to stimulate the growth of some grasses (Rorison, 1985), tropical trees (Watanabe et al., 1998), *Lespedeza bicolor* (Chen et al., 2010), and rice (Zhao et al., 2013b) when supplied with NH_4_^+^, but not when supplied with NO_3_^-^. The stimulatory effects of Al on plant growth may be related to the effects of Al to alleviate H^+^ toxicity (Kinraide et al., 1992). Thus, NH_4_^+^ alleviates Al toxicity, and Al enhances NH_4_^+^ utilization.

**Table 2:** Summary of NH_4_^+^ effects on plant Al tolerance relative to NO_3_^-^: (+) enhancement, (-) decrease, and (0) no change.

Taxon	Effects	Reference
*Holcus lanatus*	+	McCain and Davies, 1983
*Deschampsia flexuosa, Holcus lanatus*, *Bromus erectus*	+	Rorison, 1985
Spruce and beech	+	Van Praag et al., 1985^a^
*Glycine max*	+	Klotz and Horst, 1988a,b
*Secale cereal*, *Lupinus luteus*	+	Grauer and Horst, 1990
*Pinus rigida*	+	Cumming, 1990^a^; Cumming and Weinstein, 1990^a^; Schier and McQuattie, 1999^a^
*Triticosecale*	+	Antunes and Antonieta Nunes, 1997; Domingues, 2010
*Melastoma malabathricum, Acacia mangium*, *Melaleuca cajuputi*	+	Watanabe et al., 1998
*Oryza sativa*	+	Zhao et al., 2009, 2013b; Wang et al., 2015
*Lespedeza bicolor*	+	Chen et al., 2010
*Sorghum bicolor*	+ or -^b^	Tan et al., 1992
*Sorghum bicolor*	0	Keltjens, 1987
*Picea abies*	0	Godbold et al., 1988
*Mucuna pruriens*	0	Hairiah et al., 1994
*Triticum aestivum*	-	Fleming, 1983; Taylor and Foy, 1985a,b,c


**^*a*^Study was conducted using sand culture irrigated with nutrient solutions. Studies not marked by superscript letter were conducted using hydroponic systems. ^*b*^Effect was dependent on plant genotypes.**

It is now accepted that the NH_4_^+^-induced rhizosphere acidification is the primary mechanism underlying the NH_4_^+^ enhancement of Al tolerance in plants (Zhao et al., 2009; Wang et al., 2015) (**Figure 1**). Relative to NO_3_^-^, NH_4_^+^ uptake by rice roots reduces the pH of the nutrient solution. Lower pH further decreases the number of Al-binding functional groups and enhances the positive electrical potential of the root surface (Wang et al., 2015; Liu et al., 2016). Consequently, NH_4_^+^-fed roots adsorb less Al than do NO_3_^-^-fed roots, thereby alleviating Al toxicity. The ability of NH_4_^+^ to alleviate Al toxicity was also observed under constant pH conditions (Rorison, 1985; Klotz and Horst, 1988a,b; Grauer and Horst, 1990), indicating that factors other than pH may be involved. It is possible that intermediate products of N metabolism such as nitric oxide (NO) play a role in the alleviation of Al toxicity by NH_4_^+^ (Zhao and Shen, 2013).

Several studies found that NH_4_^+^ aggravated Al toxicity, relative to NO_3_^-^ (**Table 2**), which may reflect differences in plants’ sensitivity to NH_4_^+^. Some studies on the aggravating effects of NH_4_^+^ on Al toxicity used wheat as the experimental material (Fleming, 1983; Taylor and Foy, 1985a,b,c). Wheat plants prefer NO_3_^-^ to NH_4_^+^ and are sensitive to both Al and NH_4_^+^ (**Table 1**). If wheat plants are supplied only with NH_4_^+^, then NH_4_^+^ toxicity may occur and may be more serious than Al toxicity. Thus, NH_4_^+^ may aggravate rather than alleviate Al toxicity in wheat plants. Some sorghum genotypes showed lower Al toxicity and some showed higher Al toxicity with NH_4_^+^ relative to NO_3_^-^ N (Tan et al., 1992). Because an Al-sensitive sorghum genotype was more NH_4_^+^-sensitive than an Al-tolerant one, NH_4_^+^ toxicity probably masked Al toxicity in sorghum (Keltjens, 1987). Consequently, it is difficult to observe the NH_4_^+^ alleviation of Al toxicity in NH_4_^+^-sensitive plant species (Keltjens, 1987). Thus, plants grown in acid soils may suffer from Al toxicity accompanied by NH_4_^+^ toxicity due to poor soil nitrification.

Most studies on the effects of NH_4_^+^ and NO_3_^-^ on Al tolerance have been conducted using hydroponic experiments (**Table 2**), which might not reflect the real effects of NH_4_^+^ and NO_3_^-^ on Al tolerance. In soils, lower root rhizosphere pH will result in greater solubilization of Al ions from the soil into the rhizosphere solution, potentially increasing Al toxicity to plants. However, under nutrient solution culture, lower rhizosphere pH will only affect Al speciation (Keltjens and van Loenen, 1989). Lower pH due to NH_4_^+^ uptake by plants increases the solubilization of Al^3+^ from bulk soils into the rhizosphere solution (**Figure 1**). Nevertheless, for plant roots, more H^+^ in the rhizosphere solution can decrease Al^3+^ adsorption by roots through cation competition and increasing the positive electrical potential of the root surface. Thus, whether Al toxicity is exacerbated or alleviated by NH_4_^+^ or NO_3_^-^ may depend on the relative dominance of the effects of pH on Al desorption from soils into the rhizosphere solution and Al adsorption from the rhizosphere solution into the roots. Further studies on this topic should be conducted on soil-grown plants.

## Effects of Al on N Transformations in Soils

Although the effects of nitrification on soil pH and Al solubility are well known, less is known about the effects of Al on soil N transformations such as nitrification and ammonification. The nitrification rate is lower in acid soils than in neutral to calcareous soils (Che et al., 2015), although the reasons for this are still unclear. It is generally considered that low pH inhibits the activity of nitrifying microbes. Higher levels of soluble Al are often concomitant with lower soil pH. Soil N transformations are controlled by microbes. Most microbes are very sensitive to Al (Piña and Cervantes, 1996), while fungi are relatively more tolerant than bacteria to Al and acids (Zhao et al., 2013a, 2017). Low pH does not always result in high concentrations of active Al in soils, because Al ions can form complexes with various organic and inorganic ligands. Future research should explore the role of Al in regulating soil N transformations and in N cycle as a whole.

In a paper published almost 100 years ago (Denison, 1922), Al salts stimulated ammonifying microbes but adversely affected nitrifying bacteria. However, more recent reports showed that Al did not affect the nitrification potential and abundance of ammonia-oxidizing *amoA* gene of archaea and bacteria (Kasuga et al., 2010; Lin et al., 2017). Bacterial growth was shown to gradually decrease as the pH decreased from 6.5 to 4.0 (Rousk et al., 2010), while soil exchangeable Al linearly increased as the pH decreased from 5.4 to 3.7 (Aciego Pietri and Brookes, 2008). In addition, the OTU richness and Shannon’s diversity index of both ammonia-oxidizing archaea and bacteria showed significantly negative correlation with soil pH ranging from 3.77 to 8.46 (Hu et al., 2013). Therefore, microbial growth was found to be limited at soil pHs lower than 5.4 when Al became soluble, but was limited by low pH rather than Al toxicity at pHs ranging from 6.5 to 5.4. These analyses suggested that the inhibition of soil nitrification that transformed NH_4_^+^ to NO_3_^-^ was due to acid stress rather than Al toxicity, when soil pH decreased from 6.5 to 5.4. There are several soil N transformation processes such as nitrification, denitrification, and ammonification, and different types of microbes control the different pathways of transformations. To clarify the effects of Al on soil N transformation, further studies should evaluate N transformation-related microbial populations and Al solubility under controlled conditions with variable soil pH and NH_4_^+^/NO_3_^-^ supply.

## Effects of Al on NO_3_^-^ Uptake by Plant Roots

Approximately 30 published studies have focused on the effects of Al toxicity on NO_3_^-^ uptake, and most of them found that Al inhibited NO_3_^-^ uptake (**Table 3**). Jerzykiewicz (2001) observed that an extremely high concentration of Al (5 mM) even resulted in NO_3_^-^ efflux from cucumber roots. The mechanism by which Al inhibits NO_3_^-^ uptake is still unclear, but some possible mechanisms have been proposed. In one study, a high Al concentration resulted in large amounts of Al entering the symplast of soybean roots, leading to symplastic Al concentrations that were high enough to inhibit NO_3_^-^ transport across the membrane (Lazof et al., 1994). Thus, one proposed mechanism by which Al inhibits NO_3_^-^ uptake is that intracellular Al may bind to NO_3_^-^ transporters, NO_3_^-^ metabolic enzymes, and other components of systems related to NO_3_^-^ uptake. Plant NO_3_^-^ transport involves at least three systems; the constitutive high-affinity transport system (cHATS), the inducible high-affinity transport system (iHATS), and the constitutive low-affinity transport system (cLATS) (Crawford and Glass, 1998; Miller et al., 2007). The constitutive systems function without NO_3_^-^ pretreatment, but the inducible system is stimulated by external NO_3_^-^. The cHATS has low values of both *K*_m_ (6–20 μM) and *V*_max_ (0.3–0.82 μmol g^-1^h^-1^), while the iHATS is characterized by higher *K*_m_ (20–100 μM) and *V*_max_ (3–8 μmol g^-1^h^-1^) values and is induced by exposure to NO_3_^-^ for hours to days. The cLATS functions at NO_3_^-^ concentrations above 250 μM and does not become saturated even when NO_3_^-^ concentrations are as high as 50 mM. Durieux et al. (1993) reported that Al exerted stronger effects on the inducible system than on the constitutive systems. Their results also suggested that high concentrations of Al inhibited the activity of NO_3_^-^ transporters in the inducible system rather than affected the number of NO_3_^-^ transporters (Durieux et al., 1993). Pretreatment with Al had little effect on NO_3_^-^ uptake by plants (Jarvis and Hatch, 1986; Durieux et al., 1993), and NO_3_^-^ transport quickly recovered when Al was removed from the external growth medium (Durieux et al., 1993). These results suggested that Al directly interacts with NO_3_^-^ transporters but that this interaction is reversible, leading to the inhibition of NO_3_^-^ uptake by Al.

**Table 3:** Summary of effects of aluminum on NO_3_^-^ uptake: (-) inhibition, (+) stimulation, and (0) no change.

Taxon	Al (μM)	NO_3_^-^ (mM)	Al duration	Effects	Reference
*Triticum aestivum*	111	3.5	29 days	-	Fleming, 1983
*Trifolium repens*	25–100	0.7	21 days	-	Jarvis and Hatch, 1986
*Sorghum bicolor*	55–370	0.1–14	15 h–36 days	-	Keltjens, 1987, 1988; Keltjens and van Ulden, 1987; Cambraia et al., 1989; Galvez and Clark, 1991
*Pinus rigida*	200	2–4	42 days	-	Cumming, 1990^a^
*Picea abies*	37–1483	1	14 days	-	Peuke and Tischner, 1991
*Zea mays*	5–166	0.2–0.6	1.5 h–7 days	-	Durieux et al., 1993, 1995; Calba and Jaillard, 1997; Purcino et al., 2003
*Glycine max*	80	0.3	30 m–2 h	-	Lazof et al., 1994
*Triticosecale*	185, 370	1.6–12	4–7 days	-	Antunes and Antonieta Nunes, 1997; Domingues, 2010
*Musa* spp.	78.5	1.8	40 days	-	Rufyikiri et al., 2001
*Lotus japonicus*	10^2^–10^4^	0.15	24 h	-	Pal’ove-Balang and Mistrík, 2007
*Lotus corniculatus*	10^3^	0.15	72 h	-	Pal’ove-Balang and Zelinova, 2013
*Oryza sativa*	50	2.86	24–96 h	-	Zhou et al., 2016
Broadleaf trees	600	3.5	3 h	-	Burnham et al., 2017^b^
*Camellia sinensis*	400	3.6	24 h	0	Morita et al., 1998
*Glycine max*	56	1.4	14 h	+	Klotz and Horst, 1988b
*Oryza sativa*	0–1111	0.36	65 days	+ (<185 μM Al) or - (>185 μM Al)	van Hai et al., 1989
*Hordeum vulgare*	10^2^	0.37	5 min	+	Nichol et al., 1993
*Glycine max*	0–45	0.3	72 h	+ (<10 μM Al) or - (>10 μM Al)	Rufty et al., 1995
*Cucumis sativus*	500, 10^3^, 5 × 10^3^	1	1–6 h	+ (0.5 mM Al exposure for 3 h) or - (1 mM or 5 mM Al exposure for 6 h)	Jerzykiewicz, 2001
*Quercus serrata*	10^3^	2.8	3–14 days	+	Tomioka et al., 2007


*^*a*^Study was conducted using sand culture irrigated with nutrient solutions. ^*b*^Study was conducted using soil culture. Studies not marked by superscript letters were conducted using hydroponic systems.*

The inhibition of root elongation is the main symptom of Al phytotoxicity. Root elongation was inhibited much more than NO_3_^-^ uptake in the presence of high Al concentrations in soybean (Rufty et al., 1995). The Al-inhibition of NO_3_^-^ uptake was found to be similar across different Al-tolerant soybean genotypes and different root regions (Lazof et al., 1994). The root apex is the primary target of Al toxicity to plants (Ryan et al., 1993). However, NO_3_^-^ uptake rates by corn root tips only accounted for a low percentage of NO_3_^-^ taken up by the total root system, and N in root tips was mainly derived from N adsorbed through other root regions (Lazof et al., 1992). The mechanism by which Al inhibits root elongation was suggested to differ from the mechanism of Al inhibition of NO_3_^-^ uptake in maize (Durieux et al., 1995). The results of these studies indicated that the mechanism of Al inhibition of NO_3_^-^ uptake might differ from the mechanism(s) of plant Al sensitivity and Al-inhibited root elongation, at least in maize and soybean. This should be further tested using more plant species.

The effects of Al on NO_3_^-^ uptake may depend on Al concentrations, Al exposure time, plant species, and plant genotype. Aluminum does not always affect NO_3_^-^ uptake, for example, in Al-tolerant tea trees (Morita et al., 1998) (**Table 3**). A stimulatory effect of Al on root NO_3_^-^ uptake has been observed in studies where Al was supplied at low concentrations (van Hai et al., 1989; Rufty et al., 1995; Jerzykiewicz, 2001), or for a short-term (Nichol et al., 1993; Jerzykiewicz, 2001), and/or in studies on wild plant species that prefer Al (Tomioka et al., 2007) (**Table 3**). Similar to the observed stimulatory effects of Al on NO_3_^-^ uptake, N uptake and partitioning were found to be enhanced by lower Al concentrations (20–200 μM Al) but inhibited by high Al concentrations (1000 μM Al) in defoliated grasses (Thornton, 1998). In wheat, N uptake by root tips was inhibited by Al in an Al-sensitive genotype, but stimulated in an Al-tolerant genotype (Ikeda and Yamanishi, 1999). These results suggested that low Al accumulation in plants could stimulate NO_3_^-^ uptake.

**FIGURE 2:**
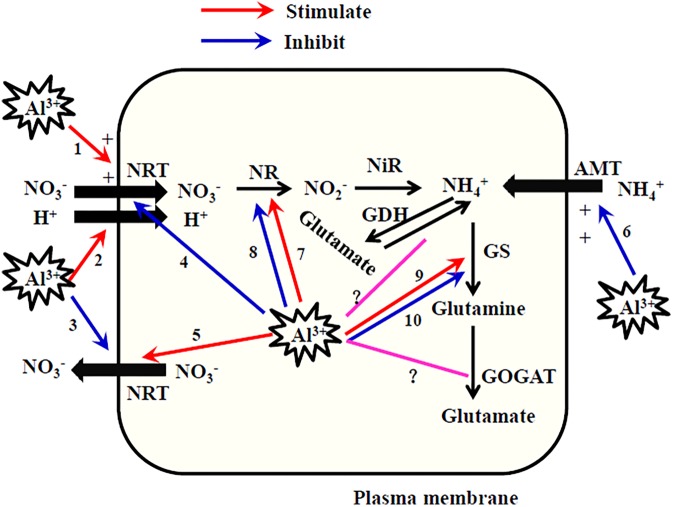
Schematic diagram of possible effects of Al on uptake and assimilation of NH_4_^+^ and NO_3_^-^ by plants. NRT, nitrate transporter; AMT, ammonium transporter; NR, nitrate reductase; NiR, nitrite reductase; GS, glutamine synthetase; GOGAT, glutamate synthase; GDH, Glutamate dehydrogenase. When plant roots accumulate low concentrations of Al in the apoplastic space, root NO_3_^-^ uptake is stimulated by apoplastic Al because of Al^3+^-increased positive electrical charge of cell surface (1), enhanced H^+^-NO_3_^-^ cotransport (2), and diminished NO_3_^-^ efflux (3). When plant roots accumulate large amounts of Al that enters the symplasm of roots, intracellular Al inhibits NO_3_^-^ uptake as Al binds to NO_3_^-^ transporter (4) and induces enhanced efflux of NO_3_^-^ (5). Al^3+^-increased positive electrical charge of cell surface results in the Al inhibition of NH_4_^+^ uptake (6). Low concentrations of Al stimulates NRA (7) because of Al-stimulated NO_3_^-^ uptake by the three ways (1, 2, and 3), while high concentrations of Al inhibits NRA (8) because of Al-inhibited NO_3_^-^ uptake by the two ways (4 and 5). Al stimulates GS activity (9) due to the binding of Al with GS while inhibits that (10) due to the inhibition of NH_4_^+^ uptake (6). The effects of Al on GOGAT and GDH are still uncertain (?).

Several possible mechanisms were suggested to be responsible for the stimulation of NO_3_^-^ uptake by low concentrations of Al (Rufty et al., 1995; Jerzykiewicz, 2001) (**Figure 2**). First, the increase in the positive electrical potential of the cell surface by Al^3+^ could facilitate the access of negatively charged NO_3_^-^ to the root cell surface. Second, Al-induced H^+^ extrusion under acid stress could increase NO_3_^-^ transport across the membrane via H^+^/NO_3_^-^ co-transport. Finally, NO_3_^-^ efflux from cells could be diminished by the binding of extracellular Al to the cell membrane if Al impairs the structural integrity of plasma membranes and alters their permeability (Cakmak and Horst, 1991). However, direct and specific evidence for each of these mechanisms is still lacking.

Rufty et al. (1995) compared experimental conditions including the Al concentration, medium pH, and calcium concentration among several papers reporting different effects of Al on NO_3_^-^ uptake. This comparative analysis suggested that pH and calcium levels, rather than Al concentrations, explained the differences in results among studies (Rufty et al., 1995). Under acid stress and low calcium levels, Al ameliorated acid stress to roots, thereby enhancing NO_3_^-^ influx into cells (Rufty et al., 1995). Further studies using carefully designed experiments should explore how pH and calcium affect the ability of Al to alter NO_3_^-^ uptake.

Based on the analyses summarized above, we present a schematic diagram to explain the mechanisms of the effects of Al on NO_3_^-^ uptake (**Figure 2**). When plant roots accumulate low concentrations of Al in the apoplastic space of roots, extracellular Al may stimulate NO_3_^-^ uptake because of an Al^3+^-induced increase in the positive electrical charge of the cell surface, enhanced H^+^-NO_3_^-^ cotransport, and diminished NO_3_^-^ efflux. When large amounts of Al enter the symplasm of roots, root NO_3_^-^ uptake is inhibited by Al because Al binds to the NO_3_^-^ transporter and enhances NO_3_^-^ efflux. We emphasize that this schematic diagram is based only on the published reports. There is still no direct evidence for these proposed mechanisms. Just as the molecular basis for N uptake has been discovered in recent years, the molecular basis of both the Al-stimulation and Al-inhibition of NO_3_^-^ transport can be explored in molecular studies on plant mutants defective in NO_3_^-^ transport.

## Effects of Al on NH_4_^+^ Uptake by Plant Roots

Various studies have reported that root NH_4_^+^ uptake was either inhibited, stimulated, or unaffected by Al (**Table 4**). However, most studies have reported inhibitory effects of Al on NH_4_^+^ uptake by plants. Nichol et al. (1993) indicated that Al treatment for 5 min suppressed the movement of cations (NH_4_^+^, Ca^2+^, and K^+^) across the plasma membrane but facilitated the movement of anions (NO_3_^-^ and phosphate). Aluminum ions may bind to the cell surface and form a positively charged layer, thereby inhibiting the adsorption of positively charged cations to the cell surface but stimulating the adsorption of negatively charged anions. Thus, similar to the mechanisms responsible for the Al stimulation of NO_3_^-^ uptake described above, the Al^3+^-induced increase in the positive electrical charge of the cell surface is responsible for the inhibition of NH_4_^+^ uptake by Al (**Figure 2**).

**Table 4:** Summary of effects of aluminum on NH_4_^+^ uptake: (-) inhibition, (+) stimulation, and (0) no change.

Taxon	Al (μM)	NH_4_^+^ (mM)	Al duration	Effects	Reference
*Oryza sativa*	0–1111	0.36	65 days	-	van Hai et al., 1989
*Sorghum bicolor*	300	0.36–3.6	2–18 days	-	Galvez and Clark, 1991
*Hordeum vulgare*	100	0.03	5 min	-	Nichol et al., 1993
*Triticum aestivum*	10, 100	2	2–3 days	-	Ikeda and Yamanishi, 1999
*Musa* spp.	78.5	0.2	40 days	-	Rufyikiri et al., 2001
*Lotus japonicus*	10^2^–10^4^	0.2	24 h	-	Pal’ove-Balang and Mistrík, 2007
*Lotus corniculatus*	10^3^	0.2	72 h	-	Pal’ove-Balang and Zelinova, 2013
*Zea mays*	166	0.2	7 days	-	Purcino et al., 2003
*Zea mays*	5–100	0.2–0.24	0.5 h–3 days	0	Durieux et al., 1993; Calba and Jaillard, 1997
*Camellia sinensis*	400	3.6	24 h	0	Morita et al., 1998
*Triticosecale*	370	0.2–1.6	4 days	0	Domingues, 2010
*Triticosecale*	185	0.8, 1.4	5–7 days	+ or 0	Antunes and Antonieta Nunes, 1997
*Sorghum bicolor*	55–370	2–4	96 h–36 days	+	Keltjens, 1987, 1988; Keltjens and van Ulden, 1987
*Glycine max*	56	1.4	14 h	+	Klotz and Horst, 1988b


**All studies used hydroponic systems*.*

In general, Al exerts a smaller negative effect on NH_4_^+^ uptake than on NO_3_^-^ uptake. In maize roots, Al reduced the uptake of both NH_4_^+^ and NO_3_^-^ but increased the uptake ratio NH_4_^+^/NO_3_^-^, indicating that NH_4_^+^ uptake was inhibited much less than NO_3_^-^ uptake by Al (Purcino et al., 2003). An Al treatment reduced NO_3_^-^ uptake but not NH_4_^+^ uptake in maize and triticale (Durieux et al., 1993; Calba and Jaillard, 1997; Domingues, 2010), while Al inhibited NO_3_^-^ uptake but stimulated NH_4_^+^ uptake in sorghum and triticale (Keltjens and van Ulden, 1987; Antunes and Antonieta Nunes, 1997). Leaf N content was increased by A1 when NH_4_^+^ was supplied but reduced by Al when NO_3_^-^ was supplied (Van Praag et al., 1985). An Al treatment reduced the NO_3_^-^ concentration but increased the free NH_4_^+^ concentration in the leaves of corn plants (Souza et al., 2016).

The studies reporting that Al stimulated root NH_4_^+^ uptake generally used N sources comprising a mixture of NH_4_^+^ and NO_3_^-^ (Keltjens, 1987, 1988; Keltjens and van Ulden, 1987; Antunes and Antonieta Nunes, 1997). Since Al inhibited NO_3_^-^ uptake in those studies, we may infer that N deficiency caused by the inhibition of NO_3_^-^ uptake might explain the stimulation of NH_4_^+^ uptake by Al. When NO_3_^-^ cannot meet the N demands of plants under Al stress, plants may take up more NH_4_^+^ in place of NO_3_^-^ to alleviate N deficiency.

## Effects of Al on NO_3_^-^ Reduction

Nitrate reductase (NR) represents the first enzymatic and rate-limiting step of NO_3_^-^ assimilation in plants. It catalyzes the reduction of nitrate to nitrite and is a substrate-inducible enzyme (Tischner, 2000). A large body of research has indicated that Al inhibits NR activity (NRA) in roots, shoots, or both (**Table 5**). Several studies reported that Al toxicity reduced NRA much more in Al-sensitive plant genotypes than in Al-tolerant ones (Foy and Fleming, 1982; Keltjens and van Ulden, 1987; Justino et al., 2006). In wheat and sorghum, Al significantly inhibited NRA in shoots rather than roots (Foy and Fleming, 1982; Keltjens and van Ulden, 1987). In contrast, Al inhibited NRA in roots rather than shoots in red spruce (Cumming and Brown, 1994). The inhibitory effect of Al on NRA may result from Al-inhibition of NO_3_^-^ uptake, as the decreased level of the substrate, NO_3_^-^, would lead to decreased NRA (Gomes et al., 1985; Keltjens and van Ulden, 1987; Keltjens, 1988; Justino et al., 2006; Pal’ove-Balang and Mistrík, 2007; Souza et al., 2016). The Al-induced decrease in NO_3_^-^ content in plants was proposed to be the main mechanism by which Al inhibits NRA, so the interaction between Al and NR may be indirect. Roots generally accumulate more Al than do shoots. However, Al significantly inhibited NRA in the shoots but not in roots of wheat and sorghum (Foy and Fleming, 1982; Keltjens and van Ulden, 1987), suggesting that a direct interaction between NR and Al is unlikely. The ratio of absorbed ^15^NO_3_^-^ to reduced ammonia-containing N remained constant with increasing Al, also suggesting an indirect effect of Al on NR (Rufty et al., 1995). However, in another study, Al inhibited the shoot NRA of sorghum, and this could not be reversed by increased NO_3_^-^ concentrations (Cambraia et al., 1989). Aluminum decreased NO_3_^-^ accumulation in cucumber roots and maize leaves but enhanced their NRA (Lidon et al., 1998; Jerzykiewicz, 2001).

**Table 5:** Summary of effects of aluminum on nitrate reductase activity: (-) inhibition, (+) stimulation, (0) no change and (N) not studied.

Taxon	Al (μM)	Al duration	Effects		Reference
				
			Root	Shoot	
*Sorghum bicolor*	50–185	5–30 days	-	-	Cambraia et al., 1989; Cruz et al., 2011^a^
*Sorghum bicolor*	55–370	48 h–24 days	0	-	Keltjens and van Ulden, 1987; Keltjens, 1988
*Oryza sativa*	160–500	5–21 days	-	-	Ganesan et al., 1993; Justino et al., 2006; Mishra and Dubey, 2011^a^
*Picea rubens*	37–370	2–42 days	-	N	Yandow and Klein, 1986
*Picea rubens*	200	10 weeks	-	0	Cumming and Brown, 1994^a^
*Pinus rigida*	200	6 weeks	-	N	Cumming, 1990^a^
*Lotus japonicus*	10^2^–10^4^	24 h	-	N	Pal’ove-Balang and Mistrík, 2007
*Zea mays*	5 × 10^4^–2 × 10^5^	15 days	N	-	Souza et al., 2016^a^
*Helianthus annuus*	100	15 days	N	-	Ruiz et al., 2007
*Hordeum vulgare*	2 × 10^3^–6 × 10^3^	6 days	N	-	Shahnawaz et al., 2017
*Triticum aestivum*	19–111	20	0	- (Al-sensitive genotype) or 0 (Al-tolerant genotype)	Foy and Fleming, 1982
*Mucuna pruriens*	110	4 weeks	N	0	Hairiah et al., 1994
*Oryza sativa*	80, 160	15 days	+ (80 μM Al) or - (160 μM Al)	+ (80 μM Al) or - (160 μM Al)	Sharma and Dubey, 2005^a^
*Zea mays*	100	15 days	- or + (dependent on genotypes and N source)	N	Mihailovic et al., 2015
*Glycine max*	56	6 h–4 days	+ or - (dependent on genotype and root distance)	N	Klotz and Horst, 1988b
*Zea mays*	10^3^	20 days	N	+	Lidon et al., 1998^b^
*Triticum aestivum*	30	3 h	+	N	Sun et al., 2014
*Glycine max*	50, 100	24 h	+	N	Wang et al., 2017
*Phaseolus vulgaris*	50	6–24 h	+	N	Wang et al., 2010
*Quercus serrata*	10^3^–2.5 × 10^3^	1 h–14 days	+	N	Tomioka et al., 2007, 2012
*Cucumis sativus*	500, 10^3^, 5 × 10^3^	24 h	+	N	Jerzykiewicz, 2001
*Triticum aestivum, Triticale hexaploidae*, and *Secale cereale*	37–370	20 days	N	+ (*Triticum aestivum*, and *Triticale hexaploidae*); - (*Secale cereale*)	Dinev and Stancheva, 1993
*Camellia sinensis*	300	14 days	+	+	Hajiboland et al., 2014
*Picea abies*	37–741	2–3 months	+ (<37 μM Al) or - (>37 μM Al)	+	Peuke and Tischner, 1991


*^*a*^study was conducted using sand culture irrigated with nutrient solutions. ^*b*^Study was conducted using vermiculite culture irrigated with nutrient solutions. Other studies not marked with superscript letters were conducted using hydroponic systems.*

In some studies, Al was found to increase NRA (**Table 5**). At low concentrations, Al stimulated NRA in spruce (<37 μM Al; Peuke and Tischner, 1991) and rice (80 μM Al; Sharma and Dubey, 2005). Aluminum stimulated NRA in the Al-preferring species *Quercus serrata* (Tomioka et al., 2007, 2012) and tea (Hajiboland et al., 2014). The production of NO mediated by NR alleviated Al toxicity in red kidney bean, wheat, and soybean by alleviating oxidative stress, where Al significantly enhanced NRA in root tips (Wang et al., 2010, 2017; Sun et al., 2014). In another study, Al more strongly promoted NRA in Al-tolerant wheat than in Al-sensitive wheat (Sun et al., 2014).

The interaction between Al and NR appears to be complex, and can be positive or negative, direct or indirect. Many environmental factors are known to modulate NRA (Tischner, 2000). In various studies, the effects of A1 on NRA depended on the plant genotype (Foy and Fleming, 1982; Keltjens and van Ulden, 1987; Justino et al., 2006; Sun et al., 2014; Mihailovic et al., 2015), plant species (Dinev and Stancheva, 1993), plant part (Foy and Fleming, 1982; Keltjens and van Ulden, 1987), medium pH (Yandow and Klein, 1986), Al levels (Peuke and Tischner, 1991; Sharma and Dubey, 2005), N source and levels (Cumming, 1990; Mihailovic et al., 2015; Gupta et al., 2016), and inoculation treatments (Cumming, 1990). Although the Al–NR interaction is complex, we can conclude that NRA is generally inhibited by high Al concentrations, and stimulated by low Al concentrations (**Figure 2**). This overall trend is similar to the effects of Al on NO_3_^-^ uptake, because NO_3_^-^ is the primary factor regulating NRA.

Further research with detailed and well-designed experiments using different plant materials is necessary to clarify the details of the interaction between NR and Al. Recently, several genes encoding NR in maize (*Zea mays*) were found to be differently modulated at the transcriptional level by Al toxicity (Cantú et al., 2016). Molecular biology techniques could be helpful to clarify the detailed mechanisms of the interaction between Al and NR as well as NO_3_^-^ uptake.

## Effects of Al on NH_4_^+^ Assimilation

In plants, NH_4_^+^ is mainly assimilated by the GS/GOGAT (glutamine synthetase/glutamate synthase) cycle, where GS catalyzes the reaction between NH_4_^+^ and glutamate to form glutamine. Glutamine subsequently combines with 2-oxoglutarate in a reaction catalyzed by GOGAT to form two molecules of glutamate (Masclaux-Daubresse et al., 2010). Glutamate dehydrogenase (GDH) is considered to be an alternative pathway to incorporate NH_4_^+^ into glutamate when plants are exposed to high NH_4_^+^ concentrations under stress. However, there is more evidence that GDH functions mainly in glutamate deamination (Masclaux-Daubresse et al., 2010). The presence of Al was shown to decrease the concentrations of NO_3_^-^-N and asparagine but increase the concentrations of amino acid-N and glutamine in the xylem sap of sorghum plants, potentially indicating that Al interferes with the synthesis and/or interconversion of N in plants (Gomes et al., 1985).

Pécsváradi’s research group reported the activating effect of the Al(III)-tartrate 1:3 complex and the Al(III)–nitrilotriacetic acid complex on the activity of GS extracted from roots and leaves of wheat (Kertész et al., 2002; Pécsváradi et al., 2009). This activating effect was attributable to the specific binding of Al to the protein chain of GS, similar to the role of Mg in activating GS activity (Pécsváradi et al., 2009). Except for those two reports (Kertész et al., 2002; Pécsváradi et al., 2009), all of the other studies summarized here reported Al inhibition of GS activity in both roots and shoots (**Table 6**). However, Al either activated, suppressed, or did not affect the activities of GOGAT and GDH (**Table 6**). The effects of Al on the activities of N-assimilating enzymes were found to vary between Al-tolerant and Al-sensitive maize varieties and depend on the N form supplied. In maize, NH_4_^+^ facilitated the Al stimulation of N assimilation in the roots of an Al-tolerant maize genotype (Mihailovic et al., 2015). Here, we suggest that Al might stimulate GS activity by binding to it, or inhibit it by limiting NH_4_^+^ uptake (**Figure 2**). However, it is difficult to draw clear conclusions about the interaction between Al and NH_4_^+^ assimilation on the basis of studies published to date. Therefore, more research is required to explore the effects of Al on these enzymes involved in NH_4_^+^ assimilation.

**Table 6:** Summary of effects of aluminum on the activities of glutamine synthetase (GS), glutamate synthase (GOGAT), and glutamate dehydrogenase (GDH): (-) inhibition, (+) stimulation, (0) no change, and (N) not studied.

Taxon	Al (μM)	Al duration	Effects		Reference
				
			Root	Shoot	
*Triticum aestivum*	10–100	5 days	GS: +	GS: +	Kertész et al., 2002; Pécsváradi et al., 2009
*Zea mays*	166	3–9 days	GS: -; NADH-GDH: +; GOGAT: 0	GS: 0; NADH-GDH: -; GOGAT: 0	Purcino et al., 2003
*Zea mays*	100	15 days	GS, NADH-GDH: (dependent on genotypes and N source)	N	Mihailovic et al., 2015
*Lotus japonicus*	10^2^–10^4^	24 h, 72 h	GS and GOGAT: -	N	Pal’ove-Balang and Mistrík, 2007, 2011
*Helianthus annuus*	100	15 days	N	GS and GOGAT: -	Ruiz et al., 2007
*Oryza sativa*	160–320	5–20	GS: -; NADH-GDH: +	GS: -; NADH-GDH: +	Mishra and Dubey, 2011^a^


*^*a*^study was conducted using sand culture irrigated with nutrient solutions. Other studies were conducted using hydroponic systems.*

## Concluding Remarks

A complex interaction between Al and N occurs in the soil–plant system. Relative to NO_3_^-^, NH_4_^+^ uptake by roots generally alleviates Al phytotoxicity under solution culture conditions, while NH_4_^+^ aggravates the solubilization of toxic Al from soils into rhizosphere solutions. Both the alleviation and aggravation effects mainly result from NH_4_^+^-induced H^+^ excretion due to NH_4_^+^ uptake by plant roots and/or soil nitrification.

Compared with the effects of N on Al, the effects of Al on N are much more complicated because N is involved in multiple physiological processes within plants. Many reports have demonstrated that Al toxicity inhibits NO_3_^-^ uptake by plant roots because Al binds to the NO_3_^-^ transporter and stimulates NO_3_^-^ efflux. In some cases, such as low Al concentrations, short-term Al exposure, and Al-preferring plants, the Al stimulation of NO_3_^-^ uptake is probably because of an increase in the positive electrical charge at the root-surface, enhanced H^+^-NO_3_^-^ cotransport, and diminished NO_3_^-^ efflux. The inhibitory effect of Al is generally smaller for root NH_4_^+^ uptake than for NO_3_^-^ uptake. Similar to the Al inhibition of NO_3_^-^ uptake, the activity of NR can be inhibited by Al treatment because of decreased internal NO_3_^-^ accumulation. Low concentrations of Al can stimulate NR activity as a result of stimulating NO_3_^-^ uptake. The effects of Al on the activities of GS, GOGAT, and GDH are still uncertain.

Despite the diverse interactions between Al and N in many studies as described above, it is clear that Al-tolerant plants generally prefer NH_4_^+^, while Al-sensitive plants prefer NO_3_^-^. This relationship between plant Al tolerance and NH_4_^+^/NO_3_^-^ preference may be the result of ecological evolution and natural selection because acid soils are characterized by a relatively higher ratio of NH_4_^+^ to NO_3_^-^ and higher concentrations of toxic Al than are neutral to calcareous soils.

Together, the results of numerous studies have suggested that the synergistic interaction between plant Al tolerance and NH_4_^+^-N nutrition may be an important strategy of plants to thrive in acid soils dominated by both toxic Al and NH_4_^+^. In addition, the Al stimulation of N uptake and assimilation can help to explain why Al stimulates plant growth in some cases.

Many studies have focused on the interactions between Al and N in plants, but the exact mechanisms underlying these interactions are still unclear. The Al–N interactions have been studied mainly at the physiological level rather than the molecular level. Physiological effects are indirectly affected by many factors and are not specific. Many genes that function in N uptake, N assimilation, and Al tolerance/toxicity have been identified (Masclaux-Daubresse et al., 2010; Ryan et al., 2011; Schroeder et al., 2013; Ma et al., 2014). The use of mutants with knocked-out or knocked-down expression of these genes could be helpful to explore the detailed mechanisms of Al–N interactions. In addition, we emphasize the importance of soil experiments for researching Al–N interactions, because the ultimate goal of understanding Al–N interactions is to improve the growth of plants in soils. Unfortunately, most studies on Al–N interactions have been conducted under solution culture conditions. As discussed above, the Al–N interactions in solutions may differ from those in soils.

How can the existing knowledge of Al–N interactions be used to improve the productivity of plants grown in acid soils? Plants need to overcome the dual limitation of Al toxicity and N deficiency in acid soils. Due to poor nitrification, acid soils have a higher NH_4_^+^ to NO_3_^-^ ratio than do neutral to calcareous soils. Large-area forest decline has been linked to both NH_4_^+^ toxicity and soil acidification, and NH_4_^+^ toxicity has become an important issue in global agriculture and ecology (Britto and Kronzucker, 2002). Symptoms of NH_4_^+^ toxicity, such as leaf chlorosis, growth suppression, and even death generally appear when the external NH_4_^+^ concentrations exceed 0.1 to 0.5 mM, depending on the plant (Britto and Kronzucker, 2002). Thus, any enhancements in plant Al tolerance in acid soils should be accompanied by improvements in plant NH_4_^+^ utilization or reduced plant NH_4_^+^ sensitivity. Although NH_4_^+^ supply generally enhances plant Al tolerance, it also increases the concentrations of toxic Al in soils and leads to potentially toxic NH_4_^+^ concentrations. How can we solve this contradiction? Which type of N fertilizer should be applied in acid soils, NH_4_^+^ or NO_3_^-^? The NO_3_^-^ fertilizers are much more expensive than NH_4_^+^ fertilizers. In addition, NO_3_^-^ is lost to water more readily than is NH_4_^+^ because NO_3_^-^ binds weakly to soil particles, which are generally negatively charged. Therefore, applying NO_3_^-^ fertilizers to acid soils appears to be impractical at the moment.

Fortunately, plants originating from acid soils are generally both Al-tolerant and NH_4_^+^-preferring. Thus, one way to increase productivity from acid soils is to breed and develop genotypes that are both Al-tolerant and NH_4_^+^-preferring. This strategy may synergistically enhance plant Al tolerance and N-use efficiency, and reduce NH_4_^+^ sensitivity and NO_3_^-^ loss. The improvement of N-use efficiency could reduce the amounts of N fertilizers applied to soils, thereby alleviating soil acidification and Al toxicity. Recently, an *in situ* ^15^N-labeling experiment showed that soluble soil Al inhibited the relative uptake of NO_3_^-^ by six tree species, potentially increasing NO_3_^-^ loss from acid soils into the surrounding water environment (Burnham et al., 2017). Thus, knowledge about Al–N interactions is important for agriculture, ecology, and the environment.

## Author Contributions

XZ wrote the manuscript. RS checked and revised the manuscript.

## Conflict of Interest Statement

The authors declare that the research was conducted in the absence of any commercial or financial relationships that could be construed as a potential conflict of interest.
